# A Rare Case of Systemic Cystic Angiomatosis Involving the Bones, Spleen, Liver, and Lungs

**DOI:** 10.7759/cureus.30414

**Published:** 2022-10-18

**Authors:** Veda Sawalgi, Dhanush Amin, Abhishek J Arora, Madhur Srivastava, Megha Uppin

**Affiliations:** 1 Department of Radiology and Imageology, Nizam's Institute of Medical Sciences, Hyderabad, IND; 2 Department of Radiodiagnosis, All India Institute of Medical Sciences, Hyderabad, IND; 3 Department of Nuclear Medicine, Nizam's Institute of Medical Sciences, Hyderabad, IND; 4 Department of Pathology and Laboratory Medicine, Nizam's Institute of Medical Sciences, Hyderabad, IND

**Keywords:** splenic nodules, fdg-pet/ct scan, vascular lesions, multi-system, rare neoplasm

## Abstract

Systemic cystic angiomatosis (SCA) is a rare disorder, usually involving the visceral organs with incidental detection during its insidious course. On radiography, it can present as multiple cystic lesions. In rare instances, it can present as mixed lesions (lytic, sclerotic) as was the case with our patient. The disease has a better prognosis than most vascular neoplasms involving the bones. We present a rare case of this disease, involving multiple organs, and presenting with an insidious onset.

## Introduction

Systemic cystic angiomatosis (SCA) is a rare disorder with multisystemic vascular involvement of the skeletal system and other organ systems, like the spleen, liver, and lungs. Classically, in the bones, SCA represents a maldeveloped vascular and/or lymphatic system, characterised by multifocal bony cysts with a honeycombed or “bubble” appearance, without aggressive osteolysis with neither peripheral soft tissue involvement nor a periosteal reaction [1]. Most of the patients present around puberty and men seem to be more frequently affected than women[2]. Visceral involvement is seen in one-third of the cases [3]. Here, we describe a case of SCA in a middle-aged female presenting with involvement of abdominal visceral organs, lungs, and bones.

## Case presentation

A 36-year-old non-diabetic, non-hypertensive female patient, who was healthy a month back, presented to us in the outpatient department with pain in the epigastric region, and upper and lower limbs pain for three weeks. It was not associated with other symptoms. The patient received a blood transfusion six months back since she was diagnosed as anemic; nothing special in her history could be elicited. The abdominal examination revealed a non-tender abdomen with gross splenomegaly without guarding or rigidity. Preliminary blood investigations showed normal hemoglobin of 12.1 gm/dl, packed cell volume (PCV) of 36.9%, total leucocyte count (TLC) of 6300 per microliter, and platelet count of 200,000 per microliter. The coagulation profile showed a normal prothrombin time of 11 seconds, an activated partial thromboplastin time of 28 seconds, and an international normalized ratio (INR) of 1.0. Preliminary tumour markers with values of serum carcinoembryonic antigen (CEA) (0.98ng/ml), CA-125 (15.87U/ml), and CA 19-9 (5.37U/ml) were within normal range.

**Figure 1:**
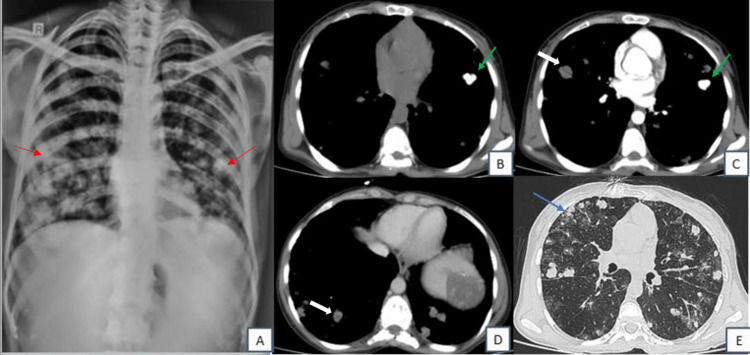
Chest radiograph postero-anterior (PA) view and axial CT chest images (plain) Multiple, well-defined radio-dense nodular lesions (red arrow) of varying sizes scattered in bilateral lung fields on chest radiograph (1A). Axial CT chest plain images show multiple solid, calcified (green arrow), and non-calcified (white arrow), parenchymal nodules of varying sizes randomly distributed in bilateral lung fields (1B, 1C). After contrast administration, non-calcified nodules (white arrow) showed minimal post-contrast enhancement (1D). A few of the nodules (blue arrow) had adjacent ground glass opacity on the lung window setting (1E).

**Figure 2:**
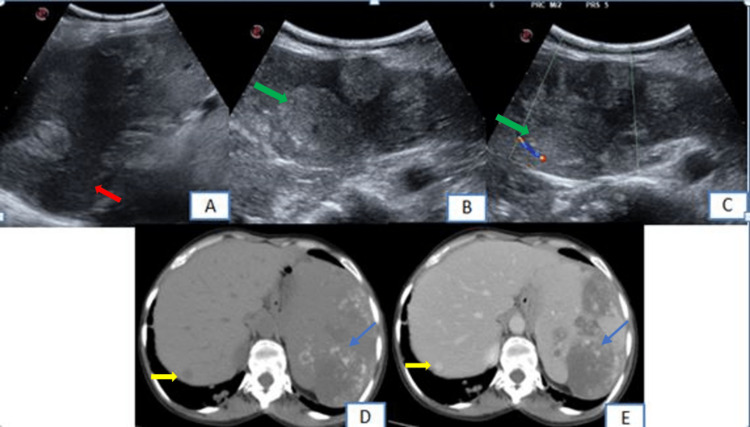
Abdominal US (plain) images and CT images showing splenic and hepatic lesions Abdominal US (plain) images (2A, 2B, 2C) showing gross splenomegaly (red arrow), with multiple, well-defined, heterogeneously hyperechoic lesions of varying sizes scattered throughout the splenic parenchyma, with no increased vascularity on Doppler evaluation (green arrow). Plain (2D) and contrast-enhanced (2E) CT images show multiple minimally enhancing lesions in the spleen (blue arrow). Hypo-dense enhancing nodule is noted in the right lobe of the liver (yellow arrow).

On imaging, the chest radiograph posteroanterior (PA) view showed multiple well-defined, radio-dense nodular lesions of varying sizes scattered in the bilateral lung fields (Figure 1A). Abdominal ultrasound (US) images showed gross splenomegaly (18.5cm) with multiple well-defined heterogeneously hyperechoic lesions of varying sizes scattered throughout the splenic parenchyma, measuring up to approximately 5 x 5 cm in the upper pole, with no increased vascularity on Doppler evaluation (Figures 2A-2C). After the US, a contrast-enhanced CT scan of the thorax and abdomen was performed which showed multiple solid calcified and non-calcified, parenchymal nodules of varying sizes randomly distributed in the bilateral lung fields (Figures 1B-1C). After contrast administration, non-calcified nodules showed minimal post-contrast enhancement (Figure 1D). Few of the nodules had adjacent ground glass opacity on lung window settings (Figure 1E). There was gross splenomegaly (18.5 cm) with multiple well-defined hypo-dense lesions showing peripheral punctate calcifications which showed minimal enhancement on post-contrast imaging. A hypo-dense nodule was noted in the right lobe of the liver which showed homogenous post-contrast enhancement (Figures 2D-2E).

**Figure 3:**
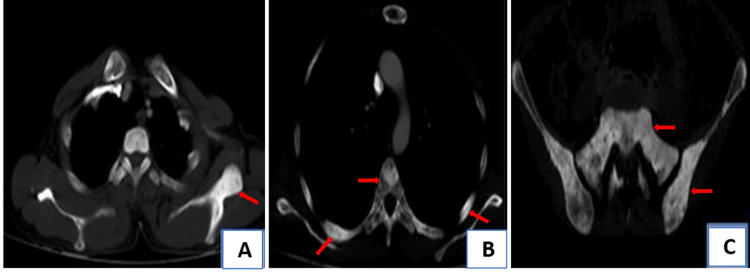
CT images in bone window setting Multiple mixed (sclerotic, lytic), mildly expansile lesions (red arrows) were seen in the left scapula (3A), bilateral ribs, and vertebral body (3B), sacrum and pelvic bones (3C).

On bone window CT images, multiple mixed (sclerotic, lytic), mildly expansile lesions were seen in the left scapula, multiple bilateral ribs, vertebral bodies, sacrum, pelvic bones, left humeral head and proximal femur. Lesions were predominantly intramedullary in location, reaching up to the cortex, however, there was no cortical break, periosteal reaction, and soft tissue component (Figure 3).

Taking into account the above findings and considering the clinical history, age, gender and imaging, primary splenic angiosarcoma with lung, liver, and skeletal metastases was thought as first differential diagnosis; lymphomatous etiology with multiple spleen, liver, lung, and skeletal deposits was considered as second differential diagnosis.

**Figure 4:**
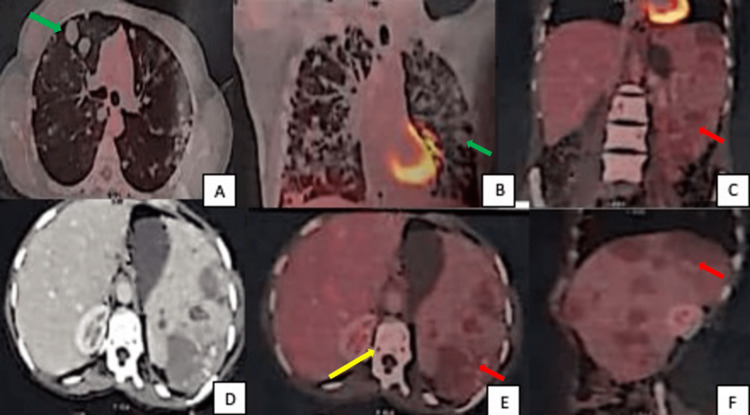
PET images showing lung, skeleton, and splenic lesions Bilateral lung fields (4A, 4B) showing multiple non-fluorodeoxyglucose (non-FDG) avid parenchymal nodules (green arrow) of varying sizes. Splenic lesions (red arrow) show minimal FDG uptake (4C, 4E, 4F). Skeletal lesions (yellow arrow) are also non-FDG avid (4E).

PET-CT was done for further evaluation which revealed bilateral lung fields showing multiple non-fluorodeoxyglucose (non-FDG) avid parenchymal nodules of varying sizes. Splenic lesions showed minimal FDG uptake, the largest measuring 5.3 x 4.2 cm with standardized uptake values (SUV) max 2.0. Skeletal lesions showed no FDG uptake. PET/CT was suggestive of metabolically inactive primary splenic lesions with extensive pulmonary and skeletal metastasis (Figure 4).

**Figure 5:**
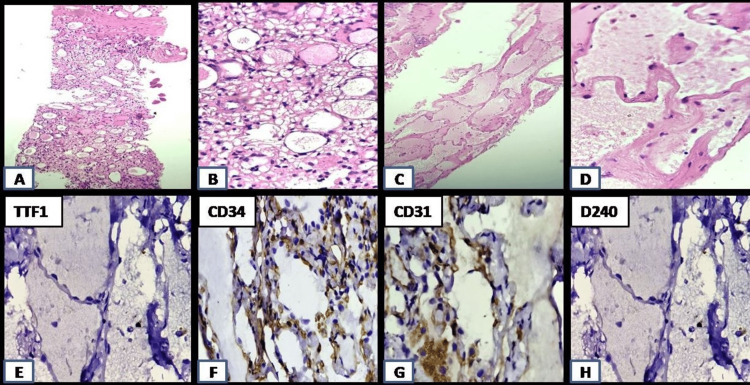
Histological sections of core needle biopsy of splenic nodule (A,B) and lung nodule (C,D) with immunohistochemistry findings of lung specimen (E-H) Histological sections of core needle biopsy of the splenic nodule (5A, 5B) and lung nodule (5C, 5D) showing multiple small vascular channels with thin fibrous walls, lined by flattened endothelium with no evidence of atypia. Features were consistent with benign vascular neoplasm. Immunohistochemistry findings of the lung specimen showed that the lesion was thyroid transcription factor 1 (TTF-1) negative (5E), CD34 and CD31 positive (5F, 5G), and D2-40 negative (5H) in endothelial cells.

For final tissue diagnosis, patient underwent US-guided biopsy of the splenic lesion which showed multiple small vascular channels with thin fibrous walls, lined by endothelium with no evidence of atypia. Features were consistent with benign vascular neoplasm. CT-guided biopsy of the lung nodules was done for further evaluation which showed multiple small vascular channels with thin fibrous walls, lined by endothelium with focal areas of hemosiderin laden macrophages and no evidence of atypia or malignancy. The lesion was thyroid transcription factor 1 (TTF-1) negative, cluster of differentiation (CD)34 and CD31 positive, and D2-40 negative in the endothelial cells (Figure 5). Features were consistent with benign vascular neoplasm. After correlating with similar splenic lesions, angiomatosis was considered.

Our patient is presently on a monthly dose of zoledronic acid of 4 mg, and is being followed up with radiological assessment of splenic, lung, liver, and bone lesions biannually. The patient is asymptomatic at present.

## Discussion

This case depicts a rare disease in which a final diagnosis could only be reached after a biopsy; its pathophysiology remains poorly understood. It is a benign disease but follows a progressive course by involving multiple organs and can lead to catastrophic outcomes due to visceral and spinal involvement. Our patient presented in middle age, hence, a metastatic disease with multiorgan involvement was the first possibility kept as differential, and the main aim of multiple diagnostic tests was to rule out the same. A similar case reported by Kumar et al. [4]considered metastatic disease due to multi-organ involvement; however, post the biopsy, turned out to be SCA. 

In a systematic review by Najm et al. [3], only 48 patients with SCA have been reported so far. The mean age of presentation was 22 years. Spinal involvement is seen in up to 50% of patients [5]. The presentation of SCA is highly variable. Majority of the cases present with bony cysts most commonly affecting the femur and pelvic bones [6]. Most commonly patients presented with joint or back pain, however, our patient presented with pain in the epigastric region and on evaluation was found to have gross splenomegaly. On reviewing the literature, the spleen is the most common extra skeletal organ which is affected by SCA and was involved in one-fourth of the cases [7].

On MRI and CT, splenic involvement usually presents as multiple slightly enhancing nodules. In our case, splenic lesions were multiple and of varying sizes with peripheral punctate calcifications, showing mild enhancement, while the coagulation profile of the patient was normal. On PET-CT, the lesions were nonspecific with no metabolic activity, and some showed moderately increased non-pathological metabolic activity[8]. Huang et al. [9] reported a case in which a 53-year-old man who presented with back pain and proximal right femur pain showed FDG avid lesions which were initially thought as neoplastic etiology but on histologic examination, led to a diagnosis of diffuse skeletal angiomatosis. In our case, the lesions showed no significant FDG uptake. Other sites include skin and soft tissues, the thymus, kidneys, mediastinum, and mesentery. Lung and pleural involvement are uncommon. The complications include bony pains, pathological fractures, deformities, splenic rupture, and spinal cord compression [3].

On imaging, the typical skeletal lesions of SCA appear as well-defined intramedullary cysts with preservation of cortical bone surrounded by a sclerotic rim without any periosteal reaction and few showing honeycomb appearance [1]. In our case, multiple lytic sclerotic lesions involving both axial and appendicular skeleton were noted. Few of them showed expansion, however, no lesion showed cortical break, periosteal reaction, and soft tissue component. Due to the involvement of multiple bones, it mimicked bone metastases, lymphoma, multiple myeloma, Paget’s disease, and histiocytosis. On histological examination, a single layer of flattened endothelial cell-lined vascular channels is typically observed [2].

There is no confirmed treatment for SCA. However, spontaneous regression leading to cure has been reported [10]. Bisphosphonates, interferon, and radiotherapy are different treatment modalities mentioned in the literature [11,12]. In high turnover states, by inhibiting osteoclastic activity, bisphosphonates decrease osteolysis and increase mineralization [13]. By inhibiting the proliferation of endothelial cells, they exert an anti-angiogenic effect [14]. The effect of radiation therapy is difficult to assess as the course of this disease is usually static [15,16].

## Conclusions

We would like to emphasize that radiological diagnosis of SCA is challenging and requires the help of pathological and laboratory investigations to reach a final diagnosis. However, we suggest that it should be kept as a differential diagnosis in patients having lesions involving multiple organs, with imaging features as described above, in most of the radiological modalities. Thus, a high index of suspicion with careful analysis of patient history and imaging features is required to establish an accurate diagnosis.
